# Supplementary material for: L-Pampo™, a Novel TLR2/3 Agonist, Acts as a Potent Cancer Vaccine Adjuvant by Activating Draining Lymph Node Dendritic Cells
Source: Cancers (Basel). 2023 Aug 4;15(15):3978. doi: 10.3390/cancers15153978 (PMC10417701; doi:10.3390/cancers15153978)
Supplement: Supplementary file 1 [file cancers-15-03978-s001.zip › cancers-2520680-supplementary.pdf]

Supplementary Mateirals

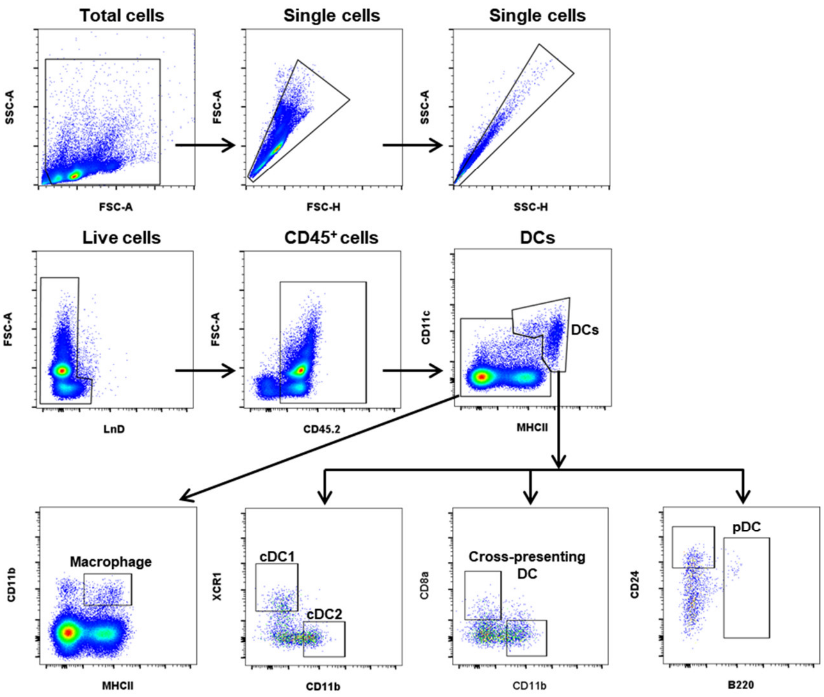

Figure S1. Gating strategy for flow cytometric analysis. Related to Figure 2. To analyze DCs and macrophages, single cells were first gated from total lymph node cells. Live cells were further gated on minimal fluorescence with a fixable viability dye. The identification of each immune populations is as follows: Immune cells (CD45$^+$); DCs (CD11c+ MHCII^{high}); macrophage (CD11c$^-$CD11b$^+$MHCII$^+$); cDC1s (CD11c+ MHCII^{high} XCR1$^+$); cross-presenting DCs (CD11c+ MHCII^{high} CD8a$^+$, CD11b$^-$); cDC2s (CD11c+ MHCII^{high} CD11b$^+$); pDCs (CD11c+ MHCII^{high} CD45B220$^+$).

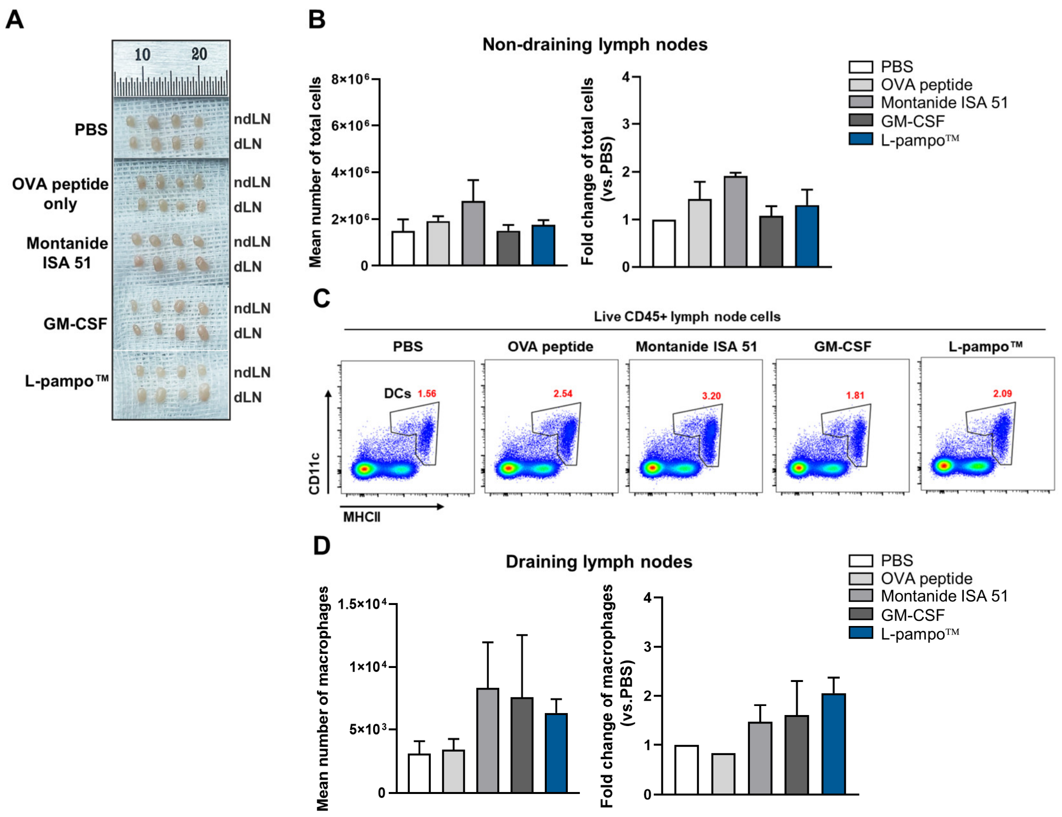

Figure S2. L-pampo™ affects DCs, but not macrophages, in draining lymph nodes (dLNs), and the impact of L-pampo™ on non-draining lymph nodes (ndLNs) is less significant. Related to Figure 2. C57BL/6 mice (total n=40; n=8/group) were s.c. injected with $OVA_{257\text{-}264}$ peptide and/or various adjuvants. including L-pampo™, Montanide ISA 51, and GM-CSF, and then inguinal lymph nodes were harvested at 24 h after injection. (**A**) Representative images of dLNs and ndLNs from the immunized mice. (**B**) The mean total $CD45^{+}$ immune cell numbers from the four ndLNs (left) and the fold changes relative to PBS (right). (**C**) The representative flow plots for the DC population in dLNs from immunized mice. Numbers in the flow plot indicate the representative frequency of the cell population (% of live $CD45^{+}$ cells) (**D**) The mean number of macrophages from the four dLNs (left) and the fold changes relative to PBS (right). Data represent mean ± SEM. Data reflect two independent experiments.

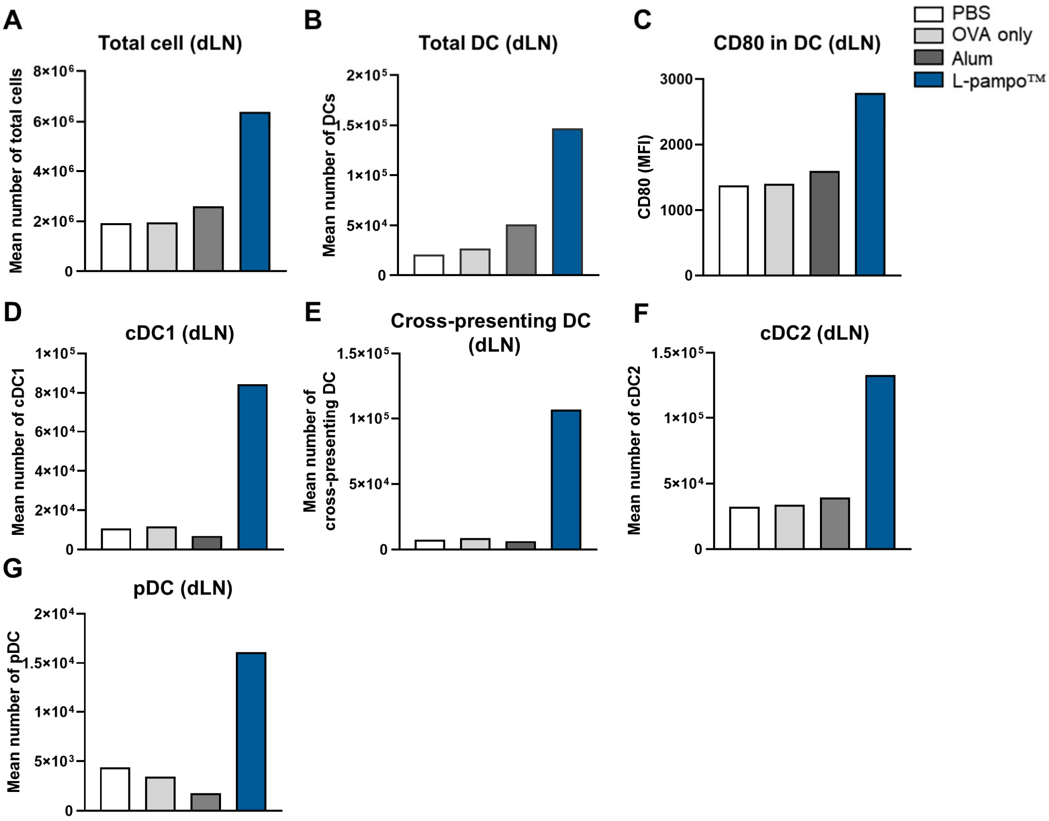

Figure S3. OVA admixed with L-pampo™ promotes DC migration and maturation, and increases DC subsets in dLNs. C57BL/6 mice (total n=32; n=8/group) were s.c. injected with OVA and/or alum and L-pampo™, and then inguinal lymph nodes were harvested at 24 h after injection. (**A**) The mean total $CD45^+$ immune cell numbers from the four draining lymph nodes. (**B**) The numbers of DC ($CD11c^+$ $MHCII^{high}$) from the four draining lymph nodes. (**C)** CD80 MFI (mean fluorescence of intensity) on DC population. (**D-G**) DC subsets in dLN were analyzed using flow cytometry and shown as the cell number. (**D**) cDC1s ($CD11c^+$ $MHCII^{high}$ $XCR1^+$) (**E**) cross-presenting DCs ($CD11c^+$ $MHCII^{high}$ $CD8a^+$) (**F**) cDC2s ($CD11c^+$ $MHCII^{high}$ $CD11b^+$) (**G**) pDCs ($CD11c^+$ $MHCII^{high}$ $CD45B220^+$).

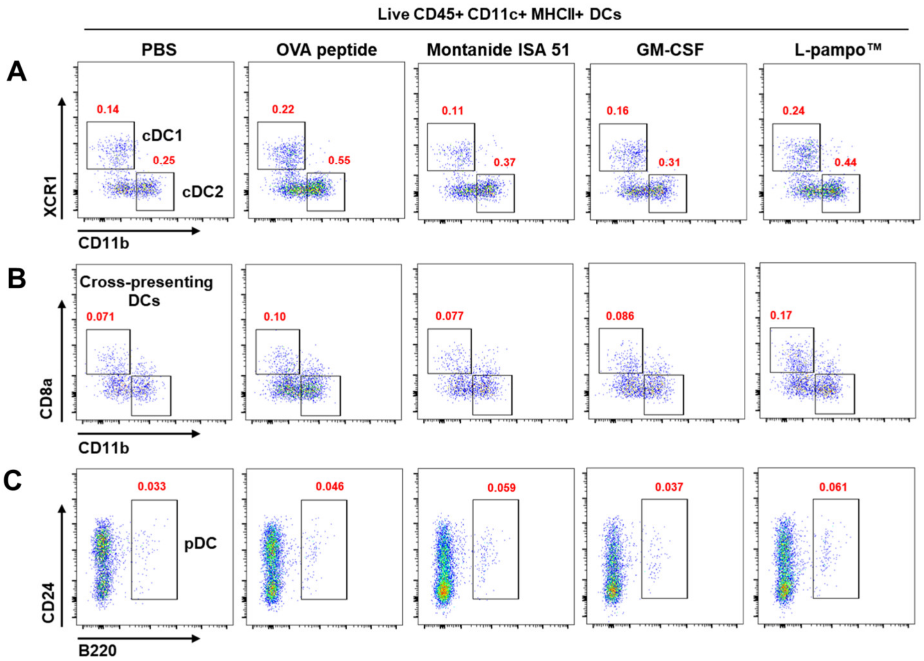

Figure S4. L-pampo™ increases DC subsets in draining lymph nodes. Related to Figure 2. C57BL/6 mice (total n=40; n=8/group) were s.c. injected with $OVA_{257\text{-}264}$ peptide and/or various adjuvants, including L-pampo™, Montanide ISA 51, and GM-CSF, and then inguinal lymph nodes were harvested at 24 h after injection. (**A-C**) DC subsets in lymph nodes were analyzed using flow cytometry (**A**) Representative flow plots of cDC1s ($CD11c^{+}$ $MHCII^{high}$ $XCR1^{+}$) and cDC2s ($CD11c^{+}$ $MHCII^{high}$ $CD11b^{+}$) (**B**) Representative flow plots of cross-presenting DCs ($CD11c^{+}$ $MHCII^{high}$ $CD8a^{+}$) (**C**) Representative flow plots pDCs ($CD11c^{+}$ $MHCII^{high}$ $B220^{+}$). Numbers in the flow plot indicate the representative frequency of the cell population (% of live $CD45^{+}$ $CD11c^{+}MHCII^{high}$ DCs).

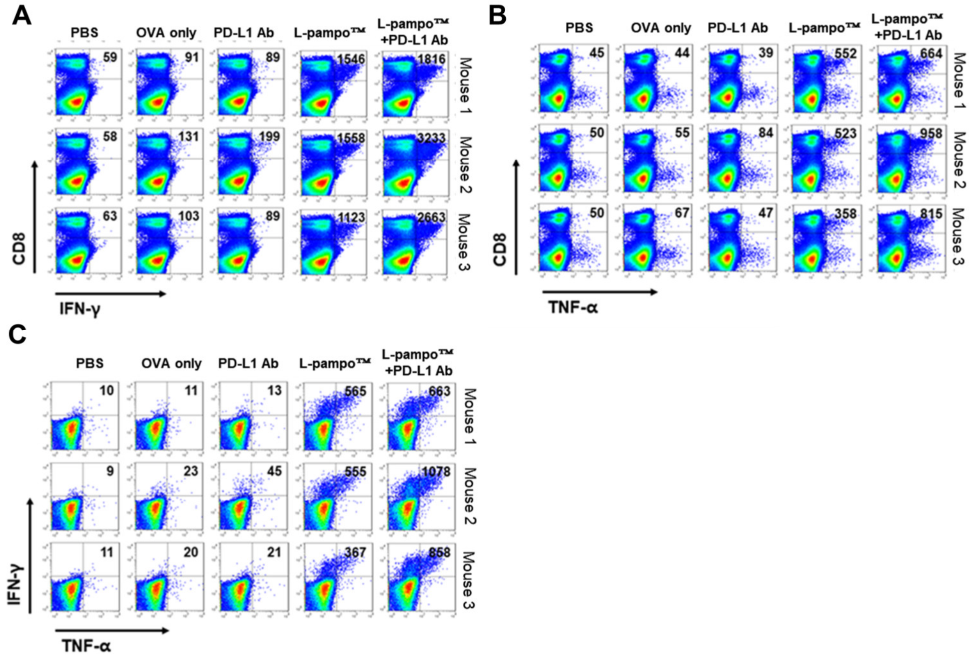

Figure S5. OVA admixed with L-pampo™ or in combination with PD-L1 inhibitor increases antigen-specific T cell immune responses. Related to Figure 5. C57BL/6 mice (total n=15; n=3/group) were subcutaneously immunized with OVA-admixed with L-pampo™ on days 0 and 7. PD-L1 antibodies were administered to immunized mice by intraperitoneal injection (i.p) on days 8, 10, and 12. Spleens were harvested on day, 14 and cytokine-producing $CD8^+$ T cells were detected using flow cytometry analyses. (**A**) Representative flow plots of OVA-specific IFN-γ-producing $CD8^+$ T cells. (**B**) Representative flow plots of OVA-specific TNF-α -producing $CD8^+$ T cells (**C**) Representative flow plots of OVA-specific IFN-γ and TNF-α producing $CD8^+$ T cells. Numbers in the flow plot indicate cytokine-producing $CD8^+$ T cell numbers.
